# A Repertory of Gonorrhœa

**Published:** 1888-06

**Authors:** 


### A Repertory of Gonorrhœa, with the Concomitant Symptoms of the Genital and Urinary Organs. By Samuel A. Kimball, M. D. Pp. 53. Price, net, $1.20. Boston: Otis Clapp & Son, 1888.

Gonorrhœa has been, perhaps, the one disease which homœopaths have treated very badly and unsuccessfully. It has been one which they have failed very frequently to cure, because they have as a rule treated the disease rather than the patient. A false idea of the disease has been borrowed from the allopaths, who consider the disease a local and a surgical one; this is crude pathology. There are two kinds of "gonorrhœa." The one is a simple urethretis, the other is true gonorrhœa; the one is easy of cure, the other often very difficult.

With this brief introduction, showing the great necessity for just such a work as this Repertory, we thank Dr. Kimball for his work, and hope it will be appreciated as it deserves—*that is, used.* The Repertory is complete in the field it treats of, is very well arranged and beautifully printed. There are two parts. Part first gives the urinary symptoms proper, and part second the symptoms of the bladder, genitals, penis, testicles, urinary discharges, gleet, chancres, buboes, warty excrescences, etc. And all this matter is carefully edited and arranged so as to be readily found, thus meeting the two vital necessities of any Repertory, which are accuracy in statement and ease in using. In compiling his work, Dr. Kimball has laboriously searched the best authorities on materia medica, and hence gives us a new as well as a reliable Repertory. Each part is arranged alphabetically, and the prominent words are clearly indicated by heavy faced type; this, with the running headings at the top of each column greatly facilitates the use of the Repertory.

Dr. Kimball offered his work as a contribution to the Bureau of Materia Medica of the I. H. A. last June; it was received with a vote of thanks by the Association and ordered to be published separately, that all might be benefited by it. The Repertory is the most useful work the Association has yet produced, and one which does it credit. If the I. H. A. could publish each year some work like this Repertory it would be doing good service for Homœopathy.

After such a promising beginning, the homœopathic profession will surely expect to hear from Dr. Kimball again in the near future.

